# 3D for the people: multi-camera motion capture in the field with consumer-grade cameras and open source software

**DOI:** 10.1242/bio.018713

**Published:** 2016-07-21

**Authors:** Brandon E. Jackson, Dennis J. Evangelista, Dylan D. Ray, Tyson L. Hedrick

**Affiliations:** 1 Department of Biological and Environmental Sciences, Longwood University, Farmville, VA 23909, USA; 2 Weapons and Systems Engineering, United States Naval Academy, Annapolis, MD 21402, USA; 3 Biology Department, University of North Carolina at Chapel Hill, Chapel Hill, NC 27599-3280, USA

**Keywords:** Videography, Photogrammetry, Kinematics, Multiple cameras, Calibration

## Abstract

Ecological, behavioral and biomechanical studies often need to quantify animal movement and behavior in three dimensions. In laboratory studies, a common tool to accomplish these measurements is the use of multiple, calibrated high-speed cameras. Until very recently, the complexity, weight and cost of such cameras have made their deployment in field situations risky; furthermore, such cameras are not affordable to many researchers. Here, we show how inexpensive, consumer-grade cameras can adequately accomplish these measurements both within the laboratory and in the field. Combined with our methods and open source software, the availability of inexpensive, portable and rugged cameras will open up new areas of biological study by providing precise 3D tracking and quantification of animal and human movement to researchers in a wide variety of field and laboratory contexts.

## Abstract (precis)

**Summary:** *Argus* is a free and open source toolset for using consumer grade cameras to acquire 3D kinematic data in field settings.

## INTRODUCTION

Many studies of biomechanics, animal behavior, evolution and ecology require that movement be quantified within complex three-dimensional (3D) environments. For example, in flight biomechanics, multi-camera high-speed videography is a staple tool for laboratory investigation of 3D animal movement (e.g. Berg and Biewener, 2010) and has led to foundational insights on the mechanics of flight (e.g. Tobalske et al., 2007), the evolution of novel locomotor strategies (e.g. Dial et al., 2008), and performance in non-steady locomotion and maneuvering (e.g. Ros et al., 2011; Warrick and Dial, 1998). However, laboratory-based studies of animal locomotion are necessarily limited in scope, and as yet, fewer studies have attempted 3D tracking in natural settings (Bahlman et al., 2013; Clark, 2009; Munk et al., 2015; Shelton et al., 2014; Sholtis et al., 2015; Theriault et al., 2014).

Many studies focus on single individuals of select species performing standardized locomotor behaviors in a confined setting. Such findings, while providing incredible insight to many aspects of animal locomotion, are therefore similarly limited in scope. Moreover, some species are more difficult than others to maintain in captivity, or require extensive training to perform tasks in a repeatable manner in an alien laboratory environment. Some behaviors (e.g. predator-prey interactions, courtship, behaviors within large groups, responses to large-scale environmental perturbations) are inherently difficult or impossible to measure within the confines of a laboratory setting. All these factors suggest that much progress in understanding locomotor behavior will come from measurement in more naturalistic settings.

A recently published protocol and associated software package allows for researchers to overcome some of the previous hurdles to multi-camera 3D videography in field settings (Theriault et al., 2014). Rather than using a carefully constructed calibration frame for calibration, Theriault et al. (2014) obtain calibration information through use of any objects in the field of view of two or more cameras, known in computer vision literature as ‘structure from motion’ (Hartley and Zisserman, 2003). Inclusion of a standardized object of known length (a ‘wand’), a scene feature such as a plumb line or water surface providing orientation to global axes, and use of the animals under study themselves aid in obtaining calibrations with low pixel and metric reconstruction errors.

The open source software implementations of Theriault et al. (2014) represent an affordable alternative to several commercially available packages; however, the workflow still assumes the use of two costly tools: (1) laboratory-grade cameras with hardware frame synchronization between multiple cameras, and (2) a MATLAB (Mathworks, Natick, MA, USA) environment. In addition to cost, laboratory-grade cameras are rarely designed for field use, are often sensitive to dust, water and mechanical forces, may be quite heavy, and often require external power and cabling that limit their deployment. Recent technological advancements and consumer demand have resulted in high-quality consumer-grade video and digital single-lens reflex (DSLR) cameras capable of moderately high-speed (≤250 frames s^−1^) video, in color and at resolutions comparable to or better than costly laboratory cameras from five years ago. Furthermore, these consumer-grade systems are designed for stand-alone operation in outdoor field settings and are capable of longer recording durations than laboratory cameras. Such consumer-grade cameras would provide a much more affordable solution for field studies of motion if two key limitations can be overcome. First, without hardware synchronization, an alternative means of synchronizing consumer-grade video cameras is needed. Second, means of coping with very wide angle, high distortion lenses is often required, especially with compact, high performance, ruggedized designs (e.g. GoPro-brand cameras).

With portability and affordability in mind, the analysis phase of video-based motion capture also may present a hurdle. At present, commercially available analysis software can be cost-prohibitive, and even current open source packages (Hedrick, 2008; Theriault et al., 2014) require MATLAB licenses or comfort with command line computer operations and programming in Python, C++ or Java. Hence, 3D motion analysis is cost- or skills-prohibitive to many investigators.

In this paper, we provide a simple workflow and user-friendly graphical tools consisting of a new open-source software package named *Argus*, aimed at overcoming the hardware and software challenges of synchronization, lens distortion and analysis of multi-camera 3D videography with consumer-grade cameras. *Argus* makes extensive use of existing open-source computer vision tools, in particular the OpenCV (v2.4.13; www.opencv.org) and sparse bundle adjustment libraries (Lourakis and Argyros, 2009) (see Open-source Python components section for a full list) for the underlying computer vision calculations, pulling these tools together in a single graphical interface targeting the needs of researchers in integrative and comparative biology. Unlike earlier efforts, *Argus* is implemented in Python and can therefore be run in a completely free, cross-platform and open source environment. Here we use the GoPro Hero4 Black series cameras as example hardware, although we have also used the techniques described here with other GoPro models, Flip MinoHD, and with Canon and Nikon DSLR cameras. Our software tools consist of graphical interfaces to access command-line routines for: (1) computing lens distortion parameters, (2) visualizing the effect of lens distortion and removing it if desired, (3) synchronizing cameras via audio channel information, (4) combining lens parameters and scene information for a full 3D calibration, and (5) digitizing video recordings of study subjects. All of our software is open source and available as both Python source code and graphical user interfaces compatible with Microsoft Windows Vista (and later), with Mac OS X (10.9 and later) and various Linux distributions. We demonstrate the use of these tools with 3D tracking of eastern carpenter bees (*Xylocopa virginica*, L. 1771) in the field. Additional materials including links to the software installation repository, installation instructions, documentation, video and written tutorials, tutorial data which includes the data presented herein, camera profiles, best-practices based on our own experience, and tips for unique situations can be found at the *Argus* website, http://argus.web.unc.edu.

### Review of 3D reconstruction

Cameras record a 2D projection of the 3D locations of points of interest. Reconstructing the 3D locations of those points from multiple videos requires precise knowledge of the cameras’ optics (the intrinsic parameters) and the cameras’ position and orientation relative to one another (the extrinsic parameters). Obtaining these parameters is typically referred to as camera calibration; a variety of methods and algorithms have been developed to perform this step (Abdel-Aziz and Karara, 1971; Lourakis and Argyros, 2009; http://www.vision.caltech.edu/bouguetj/calib_doc/), in particular see Hartley and Zisserman (2003) for a complete review. These algorithms typically seek to minimize the reconstruction error, or the distance in pixels between the observed 2D points and their theoretical 2D location given the computed 3D location. Once a calibration is available, 2D locations in two or more cameras may be used to reconstruct a 3D location, using the reprojection error as a rough measure of the quality of agreement between the 2D locations. Note that for moving objects it is crucial that the 2D locations in the separate cameras be measured at the same instant in time so that the 3D location of the object being observed is the same in both cases. Thus, a workflow such as the one below for using consumer-grade cameras to capture 3D motion in the field should include steps to quantify camera internal optics as well as field procedures for determining camera relative positions and synchronization.

1. Obtain intrinsic calibration of cameras in lab, before field use.

2. Setup cameras in the field and record setup details.

3. Make calibration and scene alignment recordings.

4. Make data recordings.

5. Make backup calibration and scene alignment recordings.

6. Analyze calibration and data videos.

In the pinhole camera model used by *Argus* the camera intrinsic parameters usually include focal length (how ‘long’ or ‘wide’ the lens is) as well as the physical size and pixel resolution of the sensor, the principal point (where the optical center of the lens is relative to the sensor), and a number of radial and tangential distortion coefficients that address image distortion relative to an ideal lens. An alternative procedure involving an omnidirectional camera model may be used for very high distortion lenses such as fisheye lenses. Intrinsic parameters are determined from video recordings of a calibration pattern swept through the field of view. For the workflow presented here, these parameters are typically obtained prior to field recordings.

The camera extrinsic parameters are the relative translation and rotation among cameras. For the work presented here, and as in (Theriault et al., 2014), these are obtained from three sources: (1) fixed points within a scene that can be matched among camera views; (2) paired points on a wand of known length, moved through the volume of interest as part of the field calibrations; and (3) other known points that can be matched among camera views, such as the study animal(s) of interest. Fixed points can be obtained by use of existing features of the site such as buildings, trees, field assistants with distinctive clothing, or purposely installed structures. These points are used to estimate an initial calibration which is then improved using sparse bundle adjustment as implemented in the SBA library (Lourakis and Argyros, 2009). Sparse bundle adjustment simultaneously optimizes the camera parameters and estimated 3D position of the observed points to minimize the disagreement between the observed 2D points and the 2D location of the computed 3D points using sparse matrix operations. Paired points separated by a known length provide scene scale and a final check to scene geometry; they can also stand in for fixed points if a sufficient number are present; that number is typically more than 50 but is highly variable and dependent on cameras and paired point positions. Wand and scene points may be identified manually using the open source software provided herein or other options such as DLTdv (Hedrick, 2008), ImageJ (NIH, Bethesda, MD) (as described in http://ww3.haverford.edu/physics-astro/Amador/links/ImageJ%20Haverflock%20Guide.docx), or any other program capable of exporting the pixel coordinates of mouse clicks or finger touches on an image.

If the objects of interest are moving, the points used for reconstruction must be taken from the same instant in time. While laboratory-grade high-speed video cameras include hardware connections for frame synchronization, consumer-grade video cameras generally lack such inputs. An alternative means of synchronization is to identify the frame or sub-frame offset between cameras and adjust the digitized points accordingly. This can be accomplished with a visual signal such as a flash, clapboard, or blinking light (e.g. Polet and Rival, 2015), or by embedding synchronization tones in the audio track (Socha et al., 2005). Here we present a precise, repeatable, and automated analysis of audio synchronization tones.

## RESULTS AND DISCUSSION

### Intrinsic calibration

**Fig. 1.**
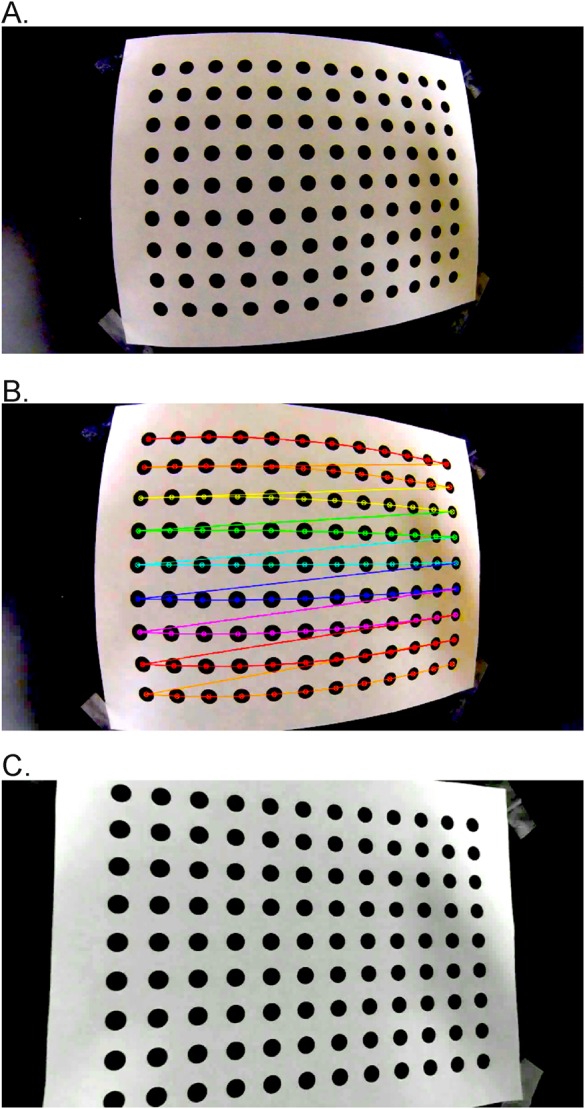
**Video undistortion operations.** Example video undistortion operations on an extracted frame from (A) a video of a dot grid pattern recorded a GoPro Hero4 camera in 1080p narrow mode showing visible optical distortion. (B) Automatic identification of the dot pattern as displayed in *Argus Patterns*; dot pattern recognition uses the OpenCV library (version 2.4). (C) The same source video, undistorted by applying distortion coefficient output of *Argus Patterns* to the source video using *Argus Dwarp*.

We filmed a 9×12 dot pattern with 2 cm spacing (Fig. 1), using a GoPro Hero4 Black camera at 120 fps, 1920×1080 narrow setting. The resulting calibration data are given in Table 1. To examine the spread in calibration values, we repeated the calibration 2000 times using patterns from 30 randomly selected frames for each replicate. The calibration with the lowest root mean squared error (rmse) value includes intrinsic parameters that fall within the 25th-75th interquartile range of all 2000 replicate calibrations, and near the median values for the best 200 calibrations, suggesting we are not in a local minimum and that the calibration parameters are significant. In the undistorted video output from *Dwarp*, the visible bulge caused by lens distortion is absent (Fig. 1C).

**Table 1.**
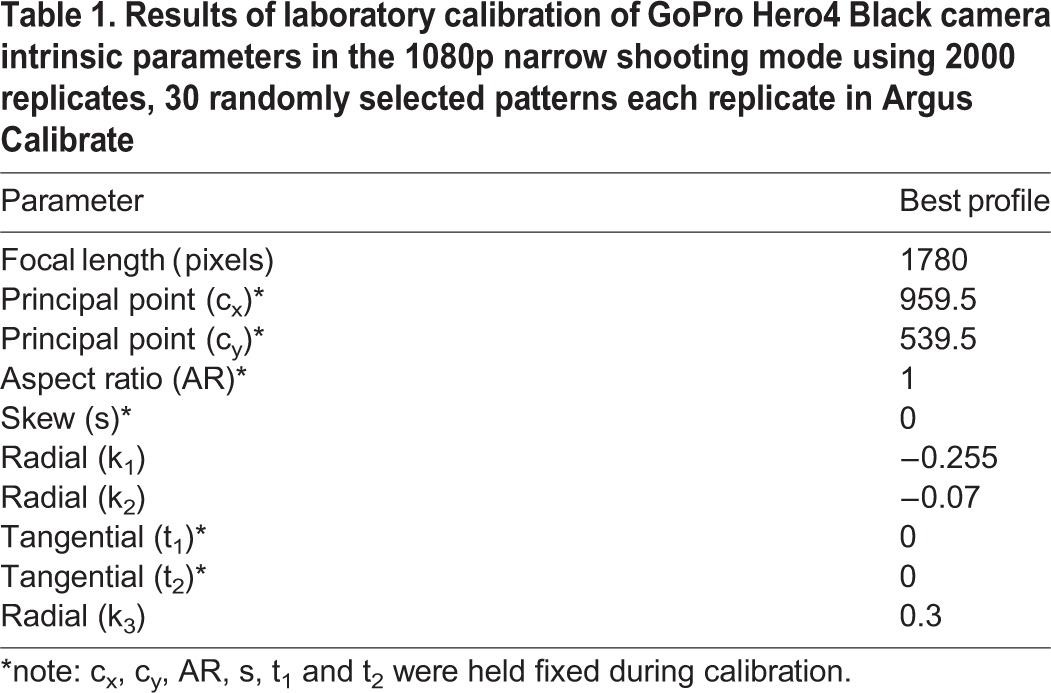
**Results of laboratory calibration of GoPro Hero4 Black camera intrinsic parameters in the 1080p narrow shooting mode using 2000 replicates, 30 randomly selected patterns each replicate in Argus Calibrate**

### Field tracking of eastern carpenter bees (*Xylocopa virginica*)

We used three GoPro Hero4 Black cameras to record eastern carpenter bees (*Xylocopa virginica*) near nest sites in Charlottesville, VA, USA. The volume of interest was approximately 3×3×6 m (l×w×h). The cameras recorded at 1080p (1920×1080 pixels) narrow field of view, at 120 fps. We used a wand built from a 6.4 mm wooden dowel, painted matte black, with polystyrene balls painted fluorescent orange (Krylon Red Glowing Orange 3101) spaced at 20 cm. Since the cameras were spaced >1 m from each other, we attached one radio (Motorola MH230R two-way radio) to the base of each camera. A fourth radio was used to send a series of tones (a built in function of the radios) to the other radios in the first 30 s of each recording for frame synchronization (audio tracks available in Supplementary information dataset 1). The wand was slowly waved through the view. Camera setup and filming of the calibration wand in the field took less than 5 min.

The wand points were automatically tracked to achieve greater than 1200 sets of paired calibration points; several bee flights were also added to the calibration to provide an additional 470 unpaired points. Two points on a hanging bird feeder were used to provide plumb-line alignment to gravity. This calibration resulted in root mean square reprojection errors of 0.94, 0.88 and 0.98 pixels for the three cameras. Variation in wand length expressed as the ratio of the standard deviation divided by the mean and multiplied by 100 was 3.6, i.e. the standard deviation was 3.6% of the mean. As a check on our 3D reconstruction, we also filmed several small rocks that we tossed through the calibrated volume. We tracked the rocks and estimated gravitational acceleration as within 2% of the expected value.

**Fig. 2.**
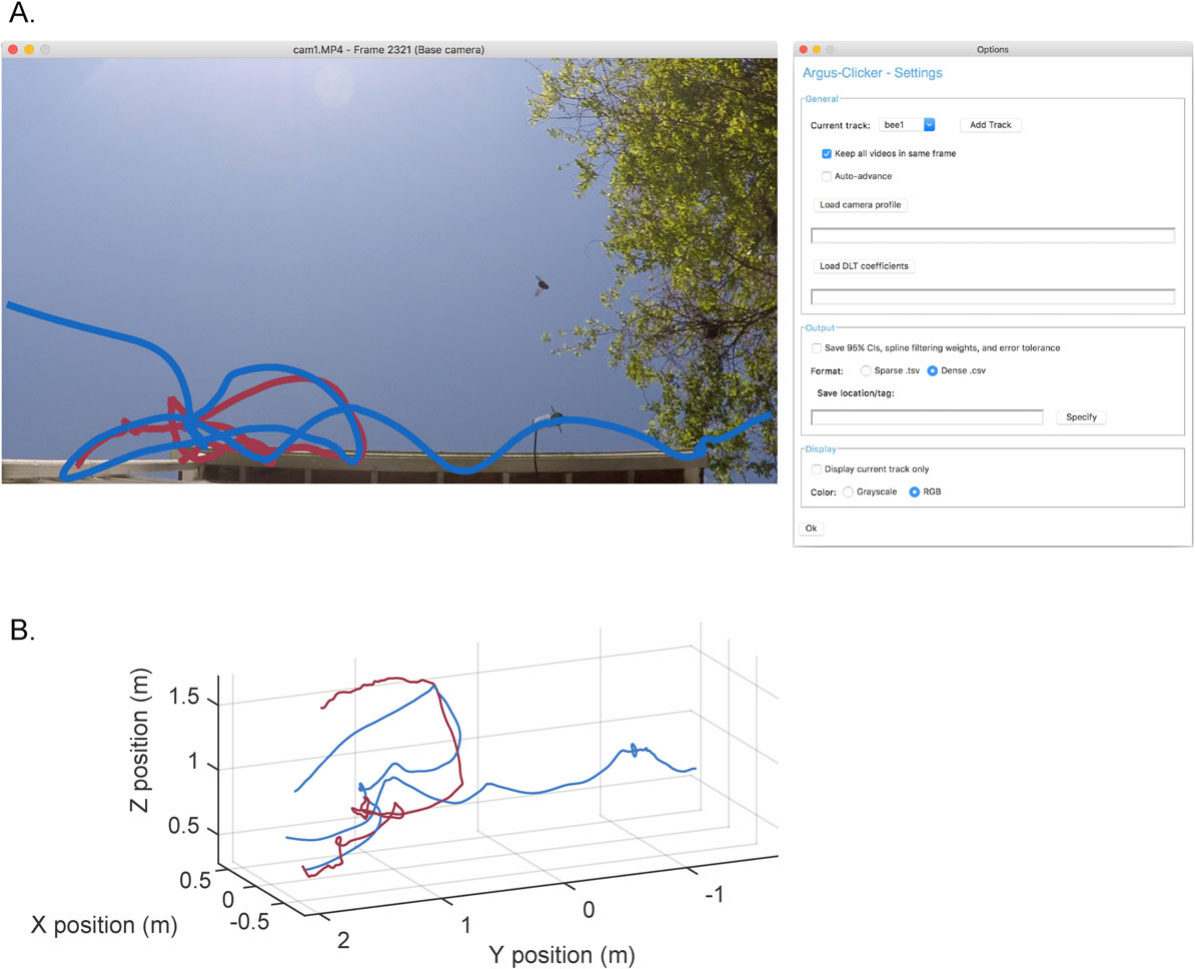
***Argus Clicker* can be used to quantify animal movement.** Trajectories from two carpenter bees in the Argus tutorial videos. (A) Trajectories in an *Argus Clicker* screenshot (modified for visual clarity here) and the options panel. (B) Trajectories in 3D coordinates arrived at by reconstructing the 2D positions shown in A and 2D positions in the other two cameras (not shown). Reconstruction requires quantification of optical distortion and camera intrinsic parameters via *Argus Patterns* and quantification of camera extrinsic parameters via *Argus Wand,* after which *Argus Clicker* can be used to generate output similar to that shown above.

**Fig. 3.**
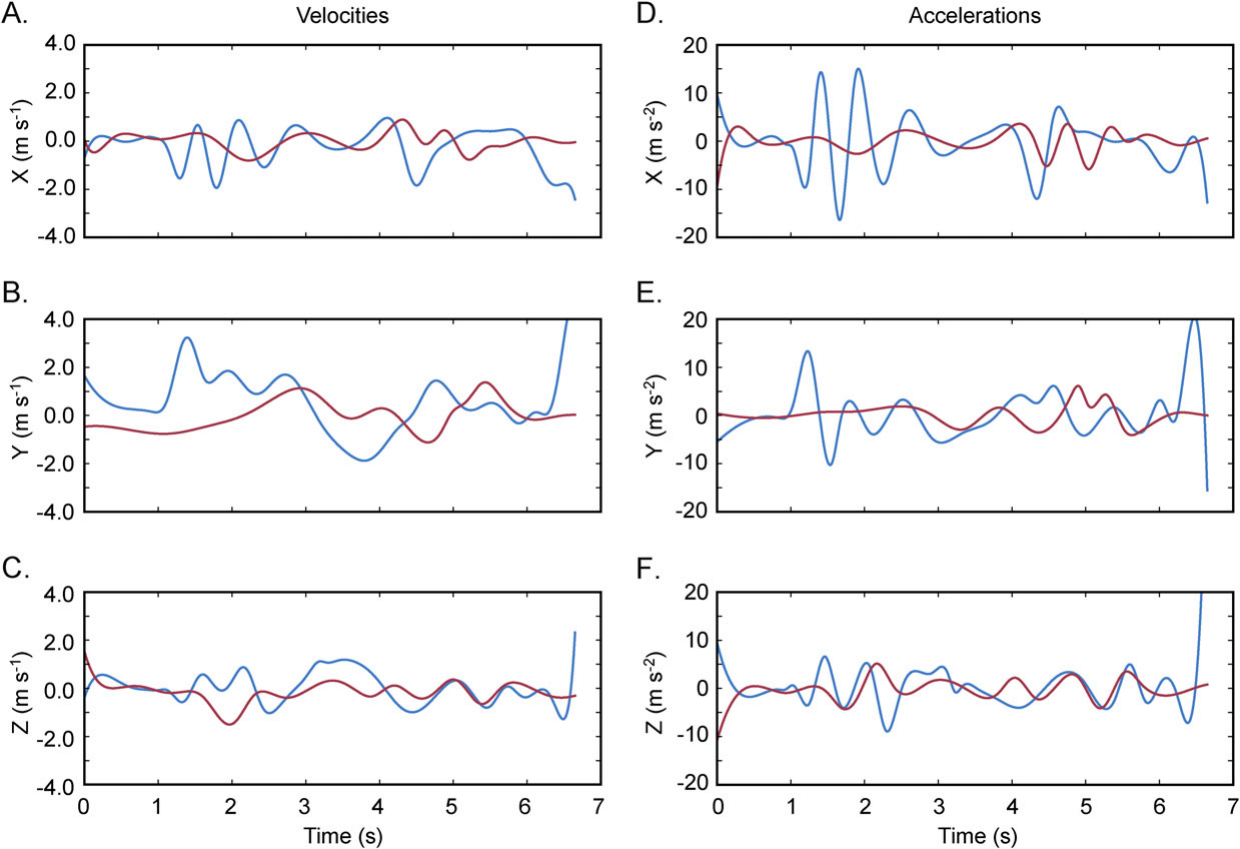
**Calculating velocities and accelerations.** Carpenter bee velocities (A-C) and accelerations (D-F) computed from the 3D trajectories shown in Fig. 2. Once the 3D trajectories have been acquired, they can be further processed to obtain many other metrics such as velocity, acceleration, radius of curvature, centripetal acceleration, distances, etc. These calculations may be performed in a wide variety of software packages including many open source options such as Python, R or Libreoffice. Here we used the SciPy package for Python to compute the derivatives and plotted them with the Matplotlib package.

In the first four minutes, we recorded over 200 flight paths of the estimated 15-20 bees near the nest sites. We manually digitized the flight paths of two bees in *Argus Clicker* (Fig. 2, and calculated 3D velocities and accelerations using custom Python scripts (Fig 3; 3D coordinates available in Supplementary information dataset 2).

### Alternatives

The multiple fixed-camera set-up described here, and analyzable in *Argus*, has one major constraint in addition to those previously discussed: the volume of interest, defined by the overlapping views of the cameras, is limited by the placement, orientation, and lens parameters of the cameras (Theriault et al., 2104). de Margerie et al. (2015) recently proposed a rotational camera prototype which records a single mounted camera's azimuth and inclination as the user pans and tilts the camera to track the movement of the target. Mirrors split the view of the single camera to provide stereography, hence distance. Because the camera can be moved, the volume of interest can be much larger than with a fixed-camera system, and it can record longer paths. However, such a system can track the position of only one animal at a time, and cannot be used to track 3D limb kinematics of the target animal. Therefore, the scientific question should direct the user to the optimal system. de Margerie et al. (2015) provide a more complete discussion of the tradeoffs between rotational and fixed-camera stereo videography systems.

*Argus Clicker* and *Wand* are free alternatives to commercially available software and MATLAB-based DLTdv (Hedrick, 2008) and easyWand (Theriault et al., 2014) packages. Currently, DLTdv and easyWand are more feature-rich in most respects than their *Argus* counterparts although they perform the same basic operations. For example, easyWand permits the user to edit camera intrinsic parameters and remove points with large errors without editing the input files; recent versions of DLTdv can split and join tracks on a per-camera basis, and the related DLTcal computes DLT coefficients using a fixed calibration frame rather than a wand. However, *Argus* development will continue to add features and the authors believe it will eventually surpass the MATLAB tools in most respects. The tools are also broadly compatible, sharing basic file formats and image coordinate systems. For example, points digitized in DLTdv can be used with *Wand* and DLT coefficients from a frame-based DLTcal calibration can be used with *Clicker*.

### Conclusion

Our goal was to provide a user-friendly open-source software package that allows for 3D reconstruction from videos recorded with consumer-grade cameras and other easily accessible components. The complete three camera package itemized in Table 2 costs less than USD $2000, and can be less expensive depending on camera models and other accessories. Once practiced, the hardware takes less than 5 min to set up and calibrate in the field, fits in a single backpack, and is durable against variable field conditions. Therefore, 3D tracking is now feasible in harsher environmental conditions than before (including under water), and where a quick deployment is necessary because animal locations are unpredictable. Additionally, because *Argus* is open-source and will run on any recent computer platform, it can be installed freely on student computers and throughout computer labs, enabling 3D tracking as part of classroom experiments.

**Table 2.**
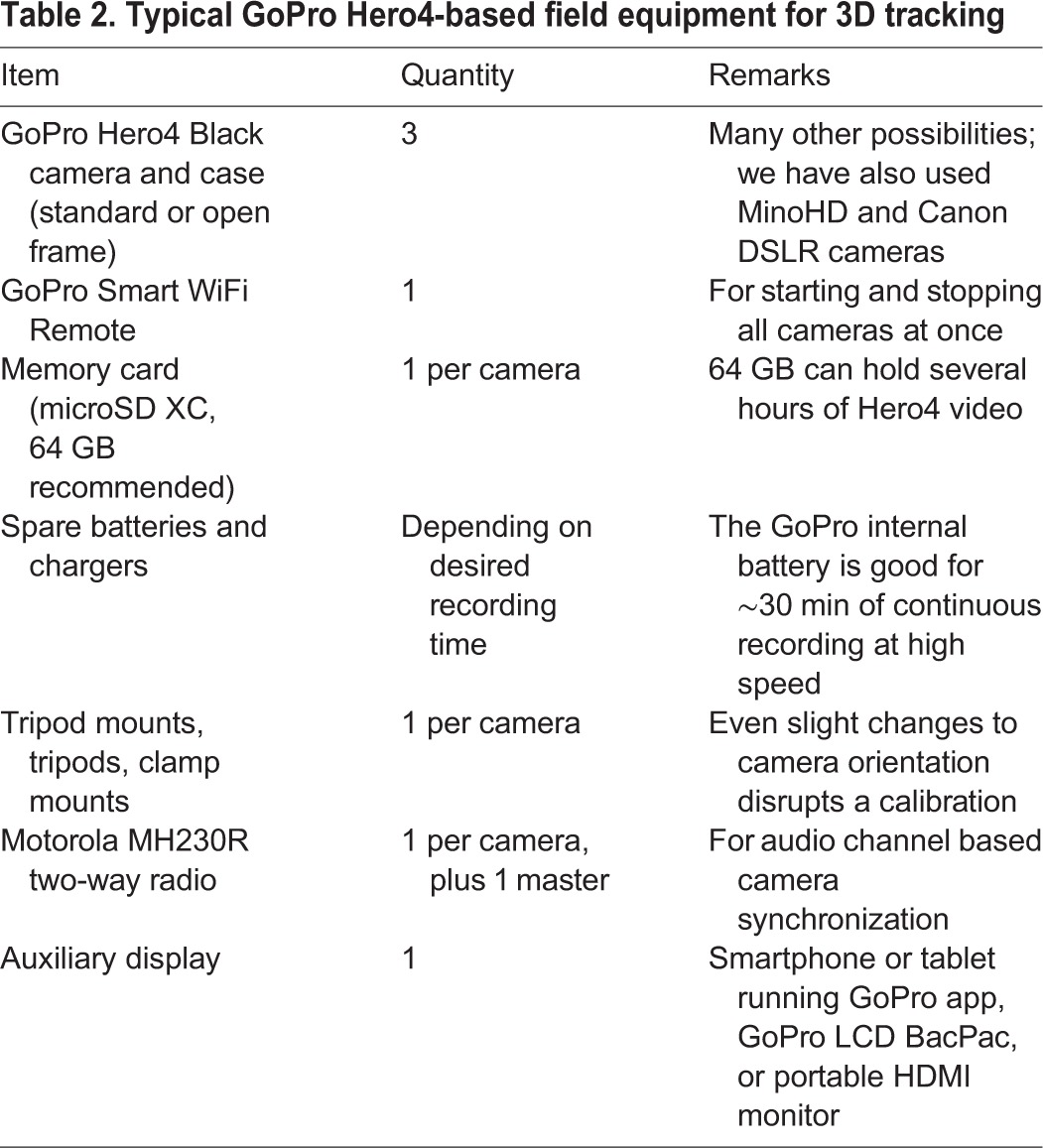
**Typical GoPro Hero4-based field equipment for 3D tracking**

## METHODS AND MATERIALS

### Cameras and equipment

We filmed using GoPro Hero4 Black cameras (GoPro, Santa Cruz, CA, USA), mounted in stock cases (see Table 2). We have also used these methods with other GoPro models, Flip MinoHD, Nikon D300S, and Canon EOS 6D, and have assisted others working with various lens-camera combinations including lab-grade video camera bodies. A typical complement of equipment for GoPro Hero4-based field 3D tracking is provided in Table 2.

### Software tools

Several software tools exist for creating extrinsic and intrinsic camera calibrations, including (Hedrick, 2008; Lourakis and Argyros, 2009; Theriault et al., 2014; http://www.vision.caltech.edu/bouguetj/calib_doc/). Here we present a simplified set of tools designed for ease of use (Table 3). The tools provided here run in a local Python environment, have graphical and command line interfaces, and are compatible with Windows Vista (and later), Mac OS X 10.9 (or later) and various Linux distributions. All code developed specifically for this project is licensed under the GNU public license version 3.0; the subcomponents use a variety of other open source licenses. The previously described DLTdv (Hedrick, 2008) and easyWand (Theriault et al., 2014) MATLAB programs provide more feature-rich implementations of point tracking and wand calibration and can take the place of different *Argus* components (*Clicker* and *Wand*, respectively) if desired. For example, DLTdv5 allows a user to split and join different digitized tracks, a feature currently not implemented in *Argus Clicker.* File formats for *Argus Clicker* and *Wand*, and for DLTdv and easyWand are largely interchangeable. The *Argus* software and documentation can be obtained from http://argus.web.unc.edu.

**Table 3.**
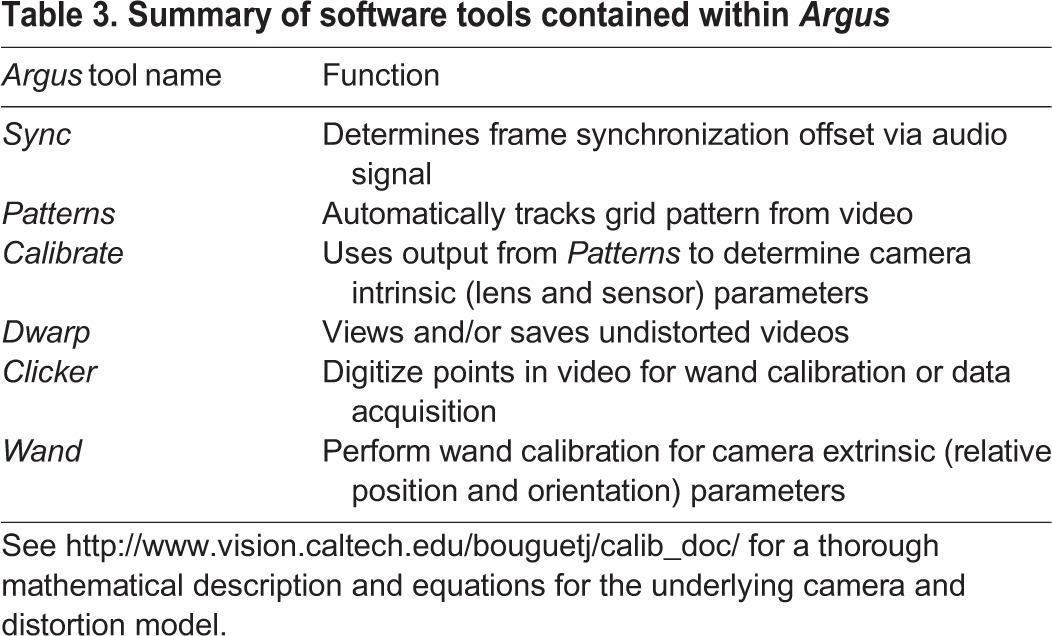
**Summary of software tools contained within *Argus***

### Laboratory calibration of camera intrinsic parameters

As part of the downloadable *Argus* software, we provide a database of camera intrinsic parameters for select camera and lens setting combinations; the database is also available separately at http://argus.web.unc.edu for use with other analysis routines (e.g. easyWand; Theriault et al., 2014). For cameras not included in the database, a laboratory calibration for camera intrinsic parameters can be obtained from *Argus*. First, a test pattern of known geometry is printed and firmly affixed to a flat surface; we typically use a high-contrast 12×9 dot pattern with 2 cm spacing (see Fig. 1, pattern available at *Argus* website).

With the camera recording at the resolution, frame rate, and field of view to be used in experiments, the pattern is moved through the field of view (or, equivalently, by moving the camera) to obtain a variety of views. An ideal calibration recording includes video frames with complete pattern views (all points are visible) at varying distances ranging from 25% to 75% of the field of view, and including all regions of the field of view. For the automatic detection routines, the orientation (landscape or portrait) of the pattern should be maintained throughout the filming; however, small rotations are desirable in order to ensure the patterns are not co-planar among different video frames. The automatic detection routines depend on sharp visible contrast of the pattern; therefore, the pattern should be well lit and should be moved slowly to reduce motion-blur.

*Argus Pattern* automatically analyzes the resulting video (see Fig. 1B) frame by frame to locate the patterns. *Argus Calibrate* uses the detected patterns to iteratively find a set of intrinsic parameters that minimizes the root mean squared error (rmse) of the reprojected points in the original pattern. Such calibration is computationally expensive and time-consuming; therefore, we designed *Argus Calibrate* to use a bootstrapping approach. The user selects the desired number of detected patterns from the calibration video, chosen randomly and non-sequentially, to include in each replicate calibration, and the number of replicate calibrations to perform. The camera profiles included herein and in *Argus* were achieved with settings of 30 frames and 2000 replicates. The intrinsic parameters saved from *Calibrate* can be used to undistort raw video using *Argus Dwarp* and are provided to downstream routines (*Argus Wand* and *Clicker*) as part of 3D reconstruction. It is important to note that such parameters will not fully remove all imperfections from a video. However, the ‘undistorted’ video output from *Dwarp*, or the inclusion of camera profiles in *Clicker*, should correct for enough distortion-induced error to provide sufficient resolution for most biological applications using these techniques.

All of the relevant *Argus* modules can work with undistorted video, and accept files containing camera profiles that are produced by *Argus Calibrate* or downloaded from the *Argus* web page. Those profiles are based on a pinhole camera model with radial and tangential distortion coefficients. We found that very high distortion fisheye lenses and wide shooting modes, such as GoPro Hero4 Black 2.7k and 1440 wide modes, are better modeled using an omnidirectional camera model (Scaramuzza et al., 2006; Urban et al., 2015). *Argus Calibrate* does not include routines for extracting omnidirectional coefficients. However*,* omnidirectional coefficients for the GoPro Hero4 wide modes are included in the *Argus* camera coefficients database and can be used within *Argus Dwarp* to undistort video that was recording with those models and settings. For calibrating other fisheye-style lenses, we recommend using the omnidirectional distortion parameter estimation software described by Urban et al. (2015) and available at https://github.com/urbste/ImprovedOcamCalib.

### Camera synchronization in the field

For cameras lacking frame exposure hardware synchronization, which includes every consumer-grade camera we tested, the audio channel recorded with each video provides an alternative means of synchronization. Even if all cameras are started by a single controller such as a GoPro Wi-Fi remote, they actually begin recording at slightly different times. The resulting offset may be several to tens of frames, and include partial frame offsets. Using the recorded audio, the video recordings can later be aligned to ensure 3D reconstructions are accomplished with pixel coordinates from the same instant in time.

Supplying clearly identifiable sounds to each camera at the same instant in time presents challenges depending on the distance between cameras. The speed of sound in air at sea level is approximately 340 ms^−1^. For cameras recording at 100 Hz spaced 10 m apart, a sound emitted near one camera may arrive at the next camera three frames later. To avoid this audio shift, we use audio synchronization tones generated by two-way radios (Motorola MH230R Talkabout), positioning one radio near each camera and holding a final master in hand. The transmission latency among devices is much less than typical camera frame rates, hence aligning audio tracks provides alignment to the video frames.

**Fig. 4.**
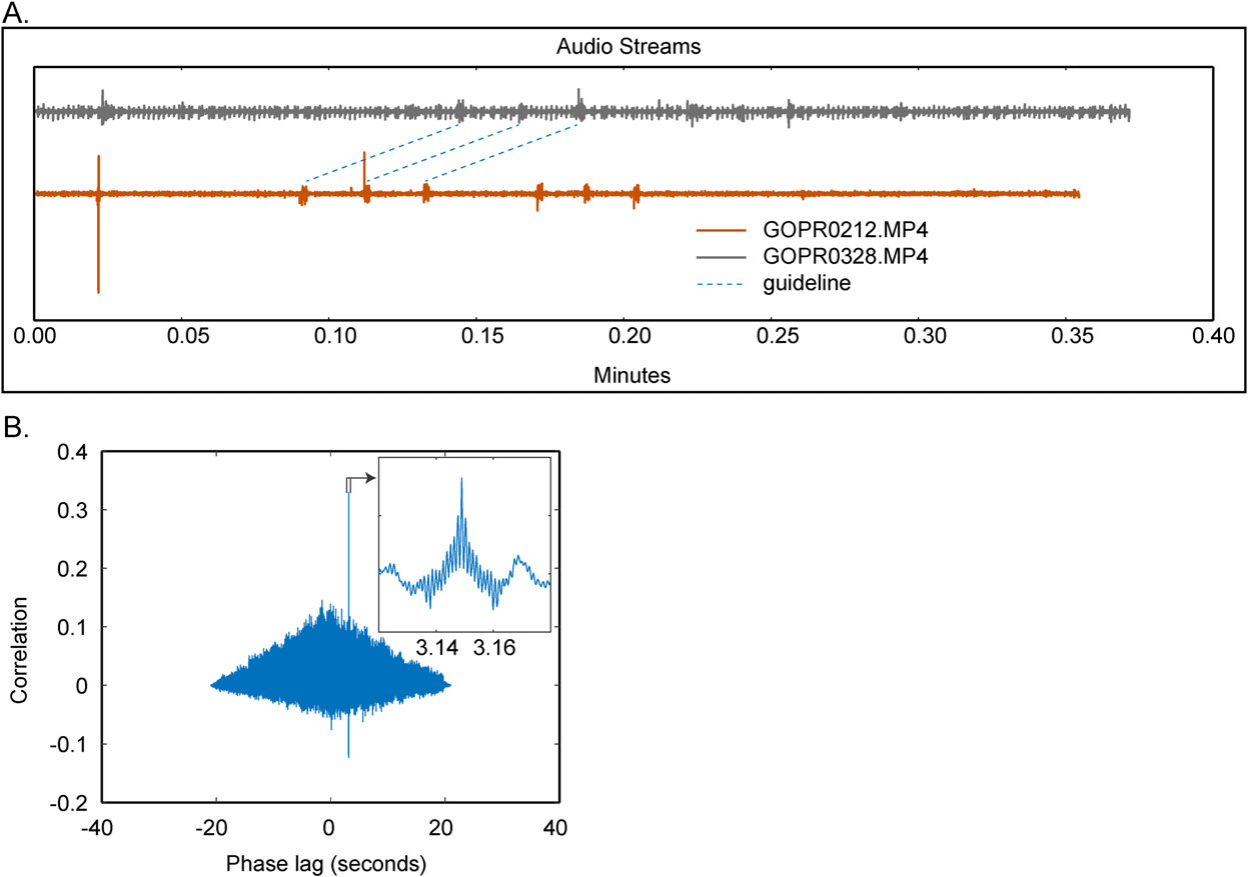
***Argus* uses cross-correlation of audio streams from the different camera files to determine the camera synchronization offset.** (A) *Argus Sync* screenshot displaying two audio streams which include six distinct calibration tones as well as substantial background noise which makes the synchronization offset difficult to determine visually. The blue dashed guidelines, added to the figure and not present in *Argus*, highlight the calibration tones and span ∼0.05 min (∼3 s). (B) Results of a cross-correlation among the two audio streams; although background noise makes the offset difficult to determine by eye, the cross-correlation identifies a distinct positive peak at ∼3.15 s; this is the actual synchronization offset between the two cameras. The cross-correlation operations are performed within *Argus Sync* and processed automatically; results are saved in a user-specified text file.

In *Argus Sync*, the offset is extracted by aligning the audio from two or more different cameras via a cross-correlation procedure, providing what we subsequently refer to as ‘soft’ synchronization. The offset is the time lag, which maximizes the cross-correlation

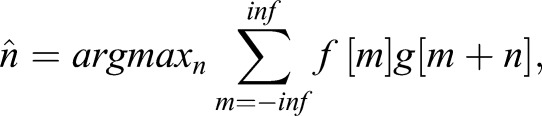
where *f* and *g* are two signals to be aligned (Fig. 4). Offsets are calculated between each camera and the first camera, and are required by *Argus Clicker* for proper alignment of videos for digitizing. Since audio frame rates are typically 44.1 or 48 kHz, much higher than video frame rates (30-240 Hz), this provides a sub-frame estimate that can either be rounded (for frame synchronization that is good enough for initial reconstruction and matching of objects moving less than half an object length per frame), or that can be used to interpolate positions for improved reconstruction accuracy if necessary.

Consumer-grade cameras may also not have their audio and video streams in perfect synchronization. This is not a concern when using a set of identical cameras because the synchronization offset is a hardware feature that should be constant within a set of like cameras. Furthermore, assuming that any audio-video synchronization differences are at a sub-frame scale, they would induce only partial frame offset errors, the effects of which are discussed below. However, if cameras of different makes and models are used some preliminary tests with an audio sync and visual signal such as a clapboard should be performed to identify any additional frame offset that needs to be added to the audio synchronization.

**Fig. 5.**
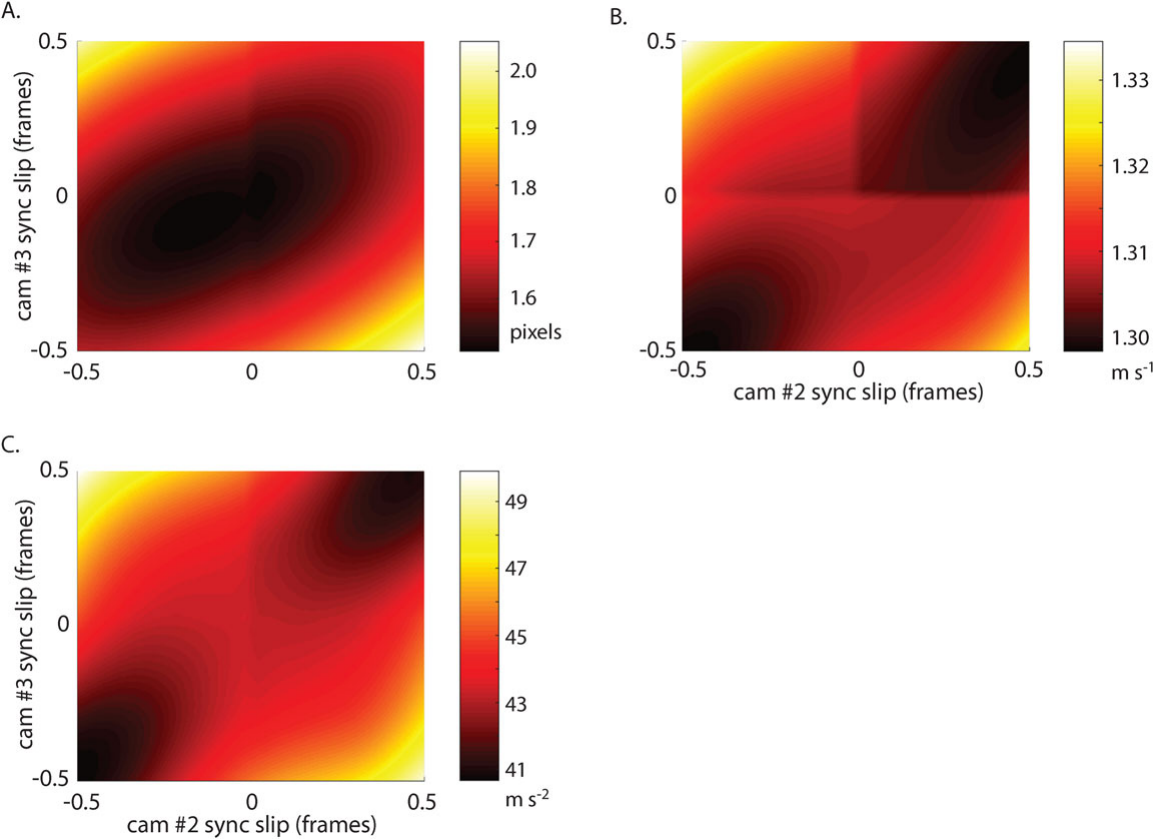
**Soft synchronization testing.** The effect of camera synchronization errors on (A) reprojection error, (B) mean speed and (C) mean acceleration magnitude for field flight data from a hummingbird (Sholtis et al., 2015). Discontinuities along the 0 slip lines are due to interaction discontinuities in the original data and linear interpolation used to artificially produce the slip.

To estimate the effects of soft synchronization on data quality, we used field-recording data of a hummingbird departing from a feeder (Sholtis et al., 2015). The original data were acquired at 100 Hz with three ‘hard’ shutter-synchronized IDT N5 cameras. Bird position was autodetected and analyzed for a variety of metrics. We resampled the original 2D position sequences across a range from −0.5 to 0.5 frames, the range of frame slip which could occur even in perfectly executed soft synchronization. The original data include gaps, which lead to slightly different results for negative and positive frame interpolation and ‘edges’ in the heat maps shown in Fig. 5. We characterized the results as mean speed and mean acceleration magnitude during the 4.5 s recording, using a 25 Hz low pass filter instead of 3 Hz as was used in the original paper. Note that with a 3 Hz filter, the simulated frame slip produces little variation in any metric, but with a 25 Hz filter frame slip leads to unrealistic acceleration magnitudes. Soft synchronization increases the reprojection error by a mean of 0.14 pixels (9%) and max 0.54 pixels (35%), affects computed velocities by a mean of 0.005 ms^−1^ (0.4%) and max 0.026ms^−1^ (2%), and affects computed accelerations by 1.36 ms^−2^ (3%) and max 6.59 ms^−2^ (15%). Note that actual error induced by soft-sync error will depend on the speed of the object of interest relative to the recording frequency of the cameras.

### Field calibration of camera extrinsic parameters

Our workflow and software implementations use three different types of scene information to create the final camera calibration: (1) unpaired points seen by two or more cameras (the background), (2) paired points separated by a known and consistent distance seen by two or more cameras (the wand), and (3) alignment points seen by two or more cameras. Successful calibrations can be obtained with different combinations of background and wand points, though only wand points are required. Alignment points are not strictly necessary, though without them the resulting 3D scene will be aligned to one of the camera views and not necessarily to gravity or other useful global frames of reference. Each of these types of scene information have associated best practices, briefly described below.

Unpaired points are typically prominent features of the scene background such as points on the horizon, corners of buildings, or marks on trees. Such points are particularly useful for calibration if they do not move, and are therefore not sensitive to camera synchronization. They also may be far from the camera (and wand) location, thus lessening the importance of measurement errors in the 2D points and improving the likelihood of the initial estimation and sparse bundle adjustment operations arriving at a high quality calibration with small wand length- and reprojection-errors.

The wand itself can be constructed in many ways, as long as it includes two discernable points separated by a known and consistent distance. Shelton et al. (2014) used the unaltered ends of meter sticks affixed to fishing lines, cast into the scene and retrieved via the fishing line; we have also experimented with arrows shot from bows, wands in the form of a bolas of known length, toy batons, and bamboo sticks attached to painter poles. Additionally, wand digitization can be aided by making the ends of the wand clearly identifiable and unique through use of high contrast stripes or colors; *Argus Clicker* includes the ability to auto-track sufficiently distinctive or high contrast points. The wand also need not be a single object. Repeated structures of identical length, such as bridge trusses or building windows, also meet the definition and can be useful in constructing calibrations. Wands should be moved through the scene, or structural ‘wands’ positioned, in such a way that the paired points as a whole are not all co-planar, fill as much of the volume of interest as possible, and present in variable orientations. Such variability ensures that wand locations provide sufficient information for a valid calibration (Hammarstedt et al., 2005; Zhang, 2004).

The 3D reconstruction from unpaired and paired points emerges with an arbitrary placement and orientation in the global field. If, for a common example, the results of 3D tracking must be interpreted in the gravitational frame of reference, the reconstruction needs to be aligned to at least the global vertical axis. A variety of alignment point possibilities are available; our tools support plumb line (two point vertical) and three axis (three points marking the origin and two cardinal axes, the third axis placed perpendicular to the plane of the other two) calibrations, but many other types have been used, ranging from alignment to gravitational acceleration measured from a falling object (Shelton et al., 2014), the surface of a body of water (Clifton et al., 2015), or local building structures (Sholtis et al., 2015).

The 3D calibration depends on marking the precise location of calibration points in each camera. If those points are moving, they must be marked at the same instant in time, which may be difficult with soft synchronization because each camera may expose the frame at slightly different times (<1/frame rate). For example, it is possible to use animal points as part of the background as described in Theriault et al. (2014); however, this should be avoided as a source of primary calibration points in soft synchronization cases unless several thousand or more points from animals moving in different directions are available. Furthermore, we recommend moving the wand slowly, or using a pose-and-hold approach where the wand is moved to each new position and held briefly for digitizing. Inclusion of inaccurately digitized or unsynchronized points may preclude computation of an accurate calibration. Soft synchronization is less of a concern when measuring the study subjects. Once an accurate 3D reconstruction is achieved, the actual position of a point of interest can be interpolated between digitized positions based on the partial frame offset provided by *Argus Sync*.

Lastly, we recommend that calibration data be obtained repeatedly, at least at the beginning and end of each data collection effort, and after any deliberate or accidental change in camera position or configuration. For example, if calibration data are recorded at the beginning and end of a multi-trial recording session, and a camera is accidentally touched between trials, the investigator can still calibrate the trials before and after the disturbance. Camera calibrations are highly sensitive to small changes in camera position, orientation, or lens settings. Through our own experience, we have found that even a slight change to camera position due to a bird landing on a GoPro case, a tripod leg settling in thick grass, or a heavy camera slowly drooping on its mount can disrupt the calibration. Therefore, the number of calibration recordings should depend on the likelihood of the cameras being disturbed in a given recording scenario. If the cameras have not been disturbed, data from multiple calibration recordings (and unpaired calibration points from any trial) can be pooled.

### Open-source Python components

*Argus* makes use of the following open-source Python components, listed here in alphabetical order. These components themselves may depend on other non-Python open source libraries such as FFmpeg (https://www.ffmpeg.org/about.html). The list of components may also change with further development of *Argus*, consult the current website documentation (http://argus.web.unc.edu) for an up-to-date list.

*audioread*, *backports-abc*, *backports.ssl-mat-hostname*, *certifi*, *cv2*, *decorator*, *imageio*, *matplotlib*, *moviepy*, *nose*, *numpy*, *pandas*, *psutil*, *Pmw*, *pygarrayimage*, *pyglet*, *pykalman*, *pyparsing*, *python-dateutil*, *pytz*, *sba*, *scipy*, *singledispatch*, *six*, *texttable*, *tornado*, *tqdm.*
